# Vaginal microbiota networks as a mechanistic predictor of aerobic vaginitis

**DOI:** 10.3389/fmicb.2022.998813

**Published:** 2022-10-20

**Authors:** Qian Wang, Ang Dong, Jinshuai Zhao, Chen Wang, Christipher Griffin, Claudia Gragnoli, Fengxia Xue, Rongling Wu

**Affiliations:** ^1^Department of Obstetrics and Gynecology, Tianjin Medical University General Hospital, Tianjin, China; ^2^Tianjin Key Laboratory of Female Reproductive Health and Eugenics, Tianjin, China; ^3^Center for Computational Biology, College of Biological Sciences and Technology, Beijing Forestry University, Beijing, China; ^4^Applied Research Laboratory, The Pennsylvania State University, State College, PA, United States; ^5^Department of Public Health Sciences, Penn State College of Medicine, Hershey, PA, United States; ^6^Division of Endocrinology, Department of Medicine, Creighton University School of Medicine, Omaha, NE, United States; ^7^Molecular Biology Laboratory, Bios Biotech Multi-Diagnostic Health Center, Rome, Italy; ^8^Center for Statistical Genetics, Department of Public Health Sciences and Statistics, The Pennsylvania State University, Hershey, PA, United States

**Keywords:** microbial interaction network, evolutionary game theory, aerobic vaginitis, quasidynamic ordinary differential equations, microbiota

## Abstract

Aerobic vaginitis (AV) is a complex vaginal dysbiosis that is thought to be caused by the micro-ecological change of the vaginal microbiota. While most studies have focused on how changes in the abundance of individual microbes are associated with the emergence of AV, we still do not have a complete mechanistic atlas of the microbe-AV link. Network modeling is central to understanding the structure and function of any microbial community assembly. By encapsulating the abundance of microbes as nodes and ecological interactions among microbes as edges, microbial networks can reveal how each microbe functions and how one microbe cooperate or compete with other microbes to mediate the dynamics of microbial communities. However, existing approaches can only estimate either the strength of microbe-microbe link or the direction of this link, failing to capture full topological characteristics of a network, especially from high-dimensional microbial data. We combine allometry scaling law and evolutionary game theory to derive a functional graph theory that can characterize bidirectional, signed, and weighted interaction networks from any data domain. We apply our theory to characterize the causal interdependence between microbial interactions and AV. From functional networks arising from different functional modules, we find that, as the only favorable genus from Firmicutes among all identified genera, the role of *Lactobacillus* in maintaining vaginal microbial symbiosis is enabled by upregulation from other microbes, rather than through any intrinsic capacity. Among *Lactobacillus* species, the proportion of *L. crispatus* to *L. iners* is positively associated with more healthy acid vaginal ecosystems. In a less healthy alkaline ecosystem, *L. crispatus* establishes a contradictory relationship with other microbes, leading to population decrease relative to *L. iners*. We identify topological changes of vaginal microbiota networks when the menstrual cycle of women changes from the follicular to luteal phases. Our network tool provides a mechanistic approach to disentangle the internal workings of the microbiota assembly and predict its causal relationships with human diseases including AV.

## Introduction

Aerobic vaginitis (AV) is an inflammatory vaginal dysbiosis that affects many aspects of health and reproduction for both pregnant and non-pregnant women worldwide (Marconi et al., 2013; Han et al., 2019; Wang et al., 2020). It is known that the occurrence of this disease is accompanied by the relative change of microbial population sizes among operational taxonomic units (OTUs). For example, the transition of Firmicutes (mainly *Lactobacillus crispatu*s and *L. iners*)-dominated microflora to Actinobacteria and Bacteroidetes-boosting microflora leads to vaginal dysbiosis and inflammation symptoms (Donders et al., 2002; Wang et al., 2020). However, results from cultivation studies show that the most common AV-related bacteria may also be represented by other types of microbes, such as *Streptococcus agalactiae*, *Staphylococcus aureus*, *Enterococcus faecalis*, coagulase-negative staphylococci (e.g., *S. epidermidis*), and *Escherichia coli* (Donders et al., 2002, 2017; Fan et al., 2013; Tang et al., 2020). In clinics, some patients met the diagnostic criteria of AV, but no underlying pathogens were identified by cultivation (Donders et al., 2002), making therapeutic intervention less effective (Donders et al., 2017). This suggest that AV is not merely related to a few aerobic microbes, but rather involves multiple bacteria that interact with each other to form intricate but well-orchestrated networks.

There is a wealth of literature on the methodological development of microbial interaction networks (Proulx et al., 2005; Faust and Raes, 2012; Vidanaarachchi et al., 2020; Matchado et al., 2021). Correlation-based networks can characterize the strength of interactions, but fail to identify the causality of interactions (Steuer et al., 2002). Bayesian networks are directed graph models, with the power to detect the causality of interactions but cannot determine the sign of the causality (Friedman et al., 2000). Most of these approaches can only reconstruct an overall network from a large number of samples, failing to characterize sample-sample heterogeneities (Kuijjer et al., 2019). Dynamic networks can capture the full information of network structure and function (Gardner et al., 2003; Sontag et al., 2004; Bansal et al., 2006; Srividhya et al., 2007; Wu et al., 2014; Chen et al., 2017), but their use in practice is impaired by the unavailability of temporal or perturbed data. Wu and team have developed a series of statistical models for inferring informative, dynamic, omnidirectional, and personalized networks (idopNetworks) from static abundance data (Chen et al., 2019, 2022; Griffin et al., 2020; Wu and Jiang, 2021). Chen et al. (2019) examined the statistical behavior of idopNetworks and their application condition. More recently, idopNetworks have been applied to predict neuroblastoma risk from a complete set of genes (Sun et al., 2020) and characterize cell crosstalk across fetal germs and their microenvironment (Wang et al., 2022), overcoming the limitation of individual genes as predictors. These networks can chart how genes are co-expressed differentially across tissues to affect human health (Wu and Jiang, 2021). Taken together, idopNetworks have emerged as a generic tool to characterize detailed topological changes in networks that describe complex systems.

In this article, we modified and implemented our network tool to reconstruct microbial interaction networks for the vaginal microbiota and reveal how microbial interactions are causally related to AV. We analyze a data set collected from a well-designed AV case-control study (Wang et al., 2020). The study monitored microbial abundance profiles from the vaginal microbiota of AV patients and healthy individuals by 16S rRNA gene sequencing. We characterized topological factors that distinguish AV-related microbial networks from healthy networks and analyzed networks changes across pH gradients. Beyond the phenomenological investigation of microbe-AV relationships based on individual microbes, our networks provide a systematic, mechanistic dissection of these relationships.

## Materials and methods

### A case-control study of vaginal microbiota

A study was initiated to assess the vaginal microbial profiles of AV patients compared with healthy individuals (Wang et al., 2020). The study includes a total of 240 participants, i.e., 80 gynecological (AV) outpatients (as the cases) at Tianjin Medical University General Hospital, China, and 160 healthy women (as the controls) who received routine examinations at the same hospital during the same period. There were strict scientific and ethical criteria for selecting these participants, as detailed in Wang et al. (2020), where the participants’ sociodemographic factors and multifaceted life behaviors were also obtained. The majority of the participants had information about the phase of their menstrual cycle; the phase-identified participants from each category (cases and controls) were classified into two groups, one in the proliferative phase (45 cases and 82 controls) and the second in the secretory phase (34 cases and 72 controls). The pH level of the vagina is associated with its healthy state; a normal vaginal pH value is between 3.8 and 4.4, whereas a pH value beyond 4.4 is abnormal for the vagina. We classify all participants including the cases and controls into three groups, one with pH = 3.8 (133 subjects), the second with pH = 4.0–4.4 (41 subjects), and the third with pH 4.6–5.4 (66 subjects). The second group is intermediate between normality and abnormality.

Wang et al. (2020) described a detailed procedure for assessing vaginal microbial profiles at operational taxonomic units (OTUs) for the participants by 16S rRNA gene sequencing. By a series of bioinformatics and statistical analysis, the microbiota in the vagina were identified at different taxonomical levels from phyla to classes to orders to families to genera to species. At each level of taxa, there exist some missing microbes whose abundance was zero. We exclude these microbes from network modeling.

### Allometric scaling quasi-dynamic ordinary differential equations

Chen et al. (2019) proposed a computational model for recovering idopNetworks from gene expression data. We modify this model to learn microbial interaction networks from static abundance data of vaginal microbes. Suppose there are *m* microbes that are measured in the vagina of each participant, regardless of its category from the cases or controls. We assume that these microbes constitute a dynamic system in which microbe-microbe interactions change from one participant to the next. Let *g*_*ji*_ denote the abundance level of microbe *j* in participant *i* and define $E_{i}=\sum_{j=1}^{m}g_{ji}$ as the habitat index (HI) of this participant. It can be seen that *g*_*ji*_ and *E*_*i*_ establish an allometric part-whole relationship, which can be quantified by a power equation, expressed as


(1) $$g_{ij}=\alpha_{j}E_{i}^{\beta_{j}}$$

where α_*j*_ and β_*j*_ are the proportionality coefficient and allometric exponent of the power equation for microbe *j* existing in participant *i*. Since *g*_*ji*_ is expressed as a function of *E*_*i*_, we use *g*_*j*_(*E*_*i*_) in place of *g*_*ji*_. Parameters α_*j*_ and β_*j*_ determine how microbe *j* changes its abundance level across participants.

We argue that the pattern of microbial interactions in a system can be interpreted through lens of evolutionary game theory. In the interactive system, a microbe attempts to maximize its abundance and fitness based on its intrinsic capacity and the strategy of other microbes that interact with it (Wu and Jiang, 2021). This attempt continues until a Nash equilibrium is reached. Allometry scaling theory in equation (Marconi et al., 2013) formulates a basis of expanding evolutionary game theory into its quasi-dynamic representation by which the pattern of how different microbes interact with each other across participants can be characterized. This representation can be expressed as a system of quasi-dynamic ordinary differential equations (qdODEs), i.e.,


(2) $$g_{j}^{\prime }\;\left(E_{i}\right)=Q_{j}\;\left(g_{j}\;(E_{i})\;;\;\phi_{j}\right)+\sum_{j^{\prime }=1,j^{\prime }\neq j}^{m}Q_{j\leftarrow j^{\prime }}\;(g_{j^{\prime }}\,\;(E_{i})\;;\;\phi_{j\leftarrow j^{\prime }})$$

with the time derivative replaced by the HI derivative, where $Q_{j}\;\left(g_{j}\;\left(E_{i}\right)\;;\;\phi_{j}\right)$ describes the (independent) abundance level of microbe *j* when it is assumed to be in isolation and $Q_{j\leftarrow j^{\prime }}\,\left(g_{j^{\prime }}\,\left(E_{i}\right);\phi_{j\leftarrow j^{\prime }}\right)$ describes the (dependent) expression level of microbe *j* regulated by microbe *j*′. The HI-varying independent abundance level can be fitted by power equation (Marconi et al., 2013) with parameters ϕ_*j*_, whereas the dependent abundance level is fitted by a non-parametric approach with parameters ϕ_*j←j’*_. We code independent components of each microbe as nodes and dependent components of each pair of microbes as edges in a (mathematical) graph (network) so that a causal network can be reconstructed. Since dependent components can be positive or negative, the networks reconstructed from equation (Han et al., 2019) can reveal the causality of microbial interactions.

### Sparsity of microbial networks through variable selection and clustering

Given that living systems are usually not fully interconnected in order to buffer against environmental stochasticity (May, 1973; Gravel et al., 2016), the microbial networks to be reconstructed should be sparse (Goyal et al., 2022; Yonatan et al., 2022). There are two strategies that can be used to ensure network sparsity. The first is to implement variable selection into a regression model built on the basis of equation (Han et al., 2019), by which a small set of the most significant microbes that link with a given microbe are chosen. Through this variable selection, *m* summations of dependent components for microbe *j*, as shown in equation (Han et al., 2019), are reduced to *d*_*j*_ summations, because the microbe *j* is found to link with only *d*_*j*_ (*d_*j*_* < < *m*) other microbes. We then solve this reduced system of qdODEs. The second strategy is to cluster all microbes into distinct modules each with a smaller number of microbes that are more strongly linked with each other than with those from different modules. Such network decomposition is consistent with developmental modularity theory, widely recognized to explain a living system’s stability and robustness in response to environmental change. We implement the procedure of the first strategy to reconstruct sparse networks for each module. Meanwhile, by taking and using the mean abundance level of all microbes within each module, we can identify the networks among modules. By linking a module-module network and its descent microbe-microbe networks, we can reconstruct multilayer and multiplex microbial idopNetworks.

### Reconstructing stable microbial interaction networks

We formulate a likelihood of microbial abundance data to solve qdODEs for network reconstruction (Wu and Jiang, 2021). Let *y*_*j*_ = (*y*_*j*_(*E*_1_),…,*y*_*j*_(*E*_*n*_)) denote a vector of abundance data for vaginal microbe *j* (*j* = 1, …, *m*) measured for *n* different samples (participants). We assume that *m* microbes interact with each other across samples in a way described by qdODE-based evolutionary game theory. The likelihood of the microbial data measured from *n* samples is written as


(3) $$L\;(y)=\prod_{i=1}^{n}f_{i}\left(y_{1};\ldots ;y_{m}\;:\;\mu_{1};\ldots ;\mu_{m},\;\Sigma \right)$$

where *f*_*i*_(⋅) is an *m*-variate longitudinal normal probability function with mean vector $\mu =\left(\mu_{1},\ldots ,\mu_{m}\right)$ and covariance matrix Σ. Explicitly, we write the mean vector as


$$\mu =\left(\mu_{1},\ldots ,\mu_{m}\right)$$

(4) $$=\left(\mu_{1}\;(E_{1}),\ldots ,\mu_{1}\,\left(E_{n}\right)\;;\;\ldots \;;\mu_{m}\;(E_{1})\;,\ldots ,\mu_{m}\,\left(E_{n}\right)\right)$$

where μ_*j* _(*E*_*i*_) is fitted by a system of qdODEs in equation (Han et al., 2019). These equations represent a fully interconnected network model, stating that each microbe is linked with all other *m* – 1 microbe. However, as mentioned above, such a full network is thought to be vulnerable, whereas a sparsely interconnected network can better buffer against stochastic perturbations (May, 1973; Gravel et al., 2016; Goyal et al., 2022; Yonatan et al., 2022). Through variable selection, we choose a small set of the *d*_*j*_ most significant microbes that are linked with a given microbe *j* as a node in the network. Thus, the full model of equation (Han et al., 2019) reduced to a reduced model in which a microbe *j* is only linked with a small number of microbes. We implement a non-parametric approach to model the independent and dependent components, specified by ODE parameters ϕ_*j*_ and ϕ_*j←j′*_ (*j* = 1, …, *m*; *j*′ = 1, …., *j* – 1, *j* + 1, …, *m*) for the reduced qdODEs. The covariance matrix has a symmetrical structure as follows:


(5) $$\Sigma =\left(\begin{array}{ccc} \Sigma_{1} & \cdots & \Sigma_{1\,m}\\ \vdots & \ddots & \vdots \\ \Sigma_{m\,1} & \cdots & \Sigma_{m} \end{array}\right)$$

where the covariance matrices for microbe *j* and between microbes *j* and *j*′ across samples are expressed as


(5A) $$\Sigma_{j}=\left(\begin{array}{ccc} \sigma_{j}^{2}\,\left(E_{1}\right) & \cdots & \sigma_{j}\,\left(E_{1},E_{n}\right)\\ \vdots & \ddots & \vdots \\ \sigma_{j}\,\left(E_{n},E_{1}\right) & \cdots & \sigma_{j}^{2}\,\left(E_{n}\right) \end{array}\right)\;$$

(5B) $$\Sigma_{j\,j^{\prime }}=\left(\begin{array}{ccc} \sigma_{j\,j^{\prime }}\,\left(E_{1}\right) & \cdots & \sigma_{j\,j^{\prime }}\,\left(E_{1},E_{n}\right)\\ \vdots & \ddots & \vdots \\ \sigma_{j\,j^{\prime }}\,\left(E_{n},E_{1}\right) & \cdots & \sigma_{j\,j^{\prime }}\,\left(E_{n}\right) \end{array}\right)$$

Since each sample represents an independent subject, it is reasonable to assume that measurement errors of the same microbes or different microbes are independent among different samples. Under this assumption, matrices in Equations 5A, 5B can be simplified as diagonal matrices in which the elements off the main diagonal are all zero. Meanwhile, we assume that residual variances for microbe *j* and residual covariances between microbe *j* and *j*′ are constant across samples. Thus, the covariance matrix of equation (Fan et al., 2013) only contains two types of parameters $\sigma_{j}^{2}$ (*j* = 1, …, *m*) and σ_*jj*′_ (*j*′ = 1, …., *j* − 1, *j* + 1, …, *m*).

By maximizing the likelihood, we implement the fourth-order Kutta-Runge algorithm in the estimation of all qdODEs that explain microbe-dependent independent and dependent abundance components and microbe-dependent residual variances and covariances. Such networks inferred from maximum likelihood estimation are stable in network topology. The maximum likelihood estimates (MLEs) of dependent abundance levels of one microbe regulated by another microbe are encapsulated in the idopNetwork, filled with bidirectional, signed, and weighted interactions and characteristic of each participant.

### Testing and comparing context-specific networks

The above procedure was used to reconstruct microbial idopNetworks in different contexts, e.g., healthy group vs. AV group, proliferative group vs. secretory group, and groups across pH gradient, etc., and allow context-known networks to be tested and compared. Consider *C* contexts of interest for network comparison. Let *y*_*cj*_ = (*y*_*cj*_(*E*_*c*1_),…,*y*_*cj*_(*E*_*cn*_*c*__)) denote a vector of the abundances of vaginal microbe *j* (*j* = 1, …, *m*) measured for *c*_*n*_ different samples from context *c* (*c* = 1, …, *C*). Under the assumption of independence in measurement error among different contexts, we formulate a joint likelihood of the microbial data measured in all *C* contexts as


(6) $$L\;(y)=\prod_{c=1}^{C}\prod_{i=1}^{n_{c}}f_{c\,i}\left(y_{c\,1};\ldots ;y_{c\,m}:\mu_{c\,1};\ldots ;\mu_{c\,m},\Sigma_{c}\right)$$

where *f*_*ci*_(⋅) is an *m*-variate longitudinal normal probability function with mean vector μ_*c*_ = (μ_*c*1_,…,μ_*cm*_) and covariance matrix Σ_*c*_ from context *c*. It is straightforward to solve the likelihood (Wang et al., 2020) by implementing the algorithmic procedure described in the previous section, including estimating the MLEs of qdODE parameters ϕ_*cj*_ and ϕ_*cj←j′*_ (*j* = 1, …, *m*; *j*′ = 1, …., *j* – 1, *j* + 1, …, *m*; *c* = 1, …, *C*) that model independent and dependent abundance components in context *c*, respectively.

Consider two different contexts, *c*_1_ and *c*_2_ (*c*_1_≠*c*_2_ = 1,…,*C*), under which microbial networks are reconstructed. To test whether the overall link structure of these microbial networks is context-dependent, we formulate the following hypotheses:

$$H_{0}\;:\;\;Q_{c_{1}j\leftarrow j^{\prime }}\;\left(g_{c_{1}j^{\prime }}\;\left(E_{i}\right)\;;\;\phi_{c_{1}j\leftarrow j^{\prime }}\right)\equiv$$

$$Q_{c_{2}j\leftarrow j^{\prime }}\;\left(g_{c_{2}j^{\prime }}\;\left(E_{i}\right)\;;\;\phi_{c_{2}j\leftarrow j^{\prime }}\right)$$

(7) $$H_{1}\;:\text{ at least one equality in the }H_{0}\text{ does not hold}$$

simultaneously for all *j* = 1, …, *m*; *j*′ = 1, …., *j* – 1, *j* + 1, …, *m*. Under the null hypothesis, the strength and direction of links between the same pair of microbes *j* and *j*′ are identical between contexts *c*_1_ and *c*_2_. We calculate the log-likelihood ratio as the test statistic using likelihood values under the null and alternative hypotheses and compare it against the critical threshold determined from permutation tests. Likewise, we can test whether a specific link between microbes *j* and *j*′ is context-dependent by formulating a similar hypothesis procedure.

We compare and test the differences in microbial network structure between the healthy and AV groups, between proliferative and secretory phases, and between different pH value levels. From these tests, we find key interaction links that determine context-dependent differences. These links can serve as a mechanistic predictor of AV risk.

## Results

### Habitat index as a predictor

**FIGURE 1:**
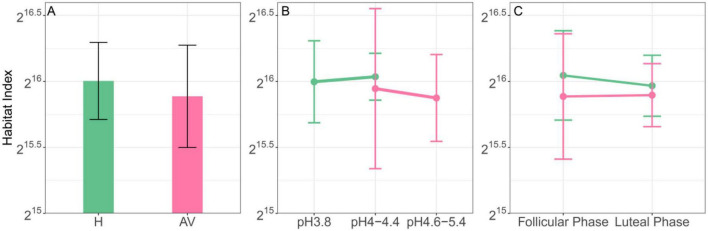
Habitat index as a predictor of AV risk. **(A)** Difference between the healthy (H) and AV groups. **(B)** Change as a function of pH values in two groups. **(C)** Difference due to the cyclic change from follicular to luteal phases for each group. Standard errors in each case are shown. Green and red lines represent H and AV groups, respectively.

The vaginal tract is viewed as an ecological habitat that is colonized by the microbiota. The sum of abundance of all microbes in a vagina, define as the habitat index (HI), may reflect the ecological carrying capacity of the vagina. We calculate the HI of each sampled vagina using Wang et al.’s (Wang et al., 2020) case-control microbial data involving AV patients (n = 80) and matched healthy subjects (H) (n = 160). We find that the HI value is slightly smaller in the AV group than in the H group (Figure 1A). Large pH values in vagina are thought to be associated with the degree of AV (Amabebe and Anumba, 2018; Lin et al., 2021). The HI decreases fairly remarkably from pH = 3.8 (healthy state) to pH = 4.6–5.4 (diseased state) (Figure 1B). At a middle range of pH (4.0–4.4) where both H and AV groups carry, the HI does not much differ between the two groups, but the AV group is considerably more variable than the H group. The HI at the proliferative phase is larger for the healthy group than AV group, but the HI of two groups tends to be convergent at the secretory phase (Figure 1C). In summary, total vagina microbes change from a healthy state to an AV state, but this change depends on the physiological states of vaginas. A more mechanistic understanding of this context-dependent change is sorely needed.

### Individual microbes as a predictor

**FIGURE 2:**
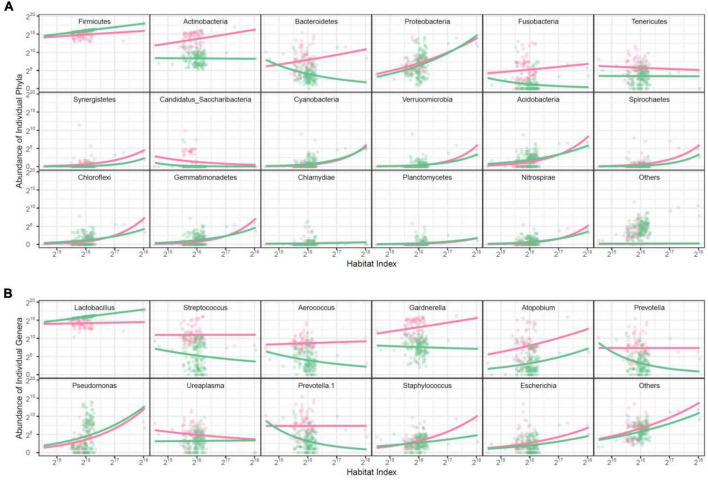
Scatterplots of abundance of individual microbes against habitat index. The change of bacterial abundance is expressed at the phylum level **(A)** and the genus level **(B)**. Dots represent a phylum or genus, whose HI-varying abundance change is fitted by the power equation. Red color for The AV group is represented in red while the healthy group is in green.

**Taxonomic microbes:** The abundance level of individual microbes establishes a part-whole relationship with HI across samples. This relationship obeys a physical principle that can be fitted by the allometric scaling power equation. As such, we can express the abundance values of individual microbes collected in discrete samples as a quasi-dynamic function of HI (Griffin et al., 2020). We choose the richest 17 identified phyla and a mix of unidentified phyla (denoted as others) for data modeling and analysis. Among all the phyla studied, only Firmicutes is more abundant and also increases its abundance with HI at a greater slope in the healthy group than in the AV group (Figure 2A). The abundance of Actinobacteria, Bacteroidetes, Fusobacteria, and Tenericutes is much richer in the AV than healthy group; the abundance of the first three phyla increases with HI in the AV group but decreases with HI in the healthy group, whereas the abundance of Tenericutes decreases its abundance with HI in both groups.

We further plot the abundance of individual genera against HI, which is also found to obey the power equation (Figure 2B). Genus *Lactobacillus* from Firmicutes has greater abundance and also increases its abundance with HI in the healthy group than in the AV group. All other genera are either much more abundant over a full range of HI in the AV and healthy group, such as *Streptococcus* and *Aerococcus* from Firmicutes and *Gardnerella*, *Atopobium*, and *Prevottella* from the other phyla, or are consistent between the two groups. Overall, only *Lactobacillus* is a favorable genus, contributing to maintaining a healthy vaginal ecosystem.

**FIGURE 3:**
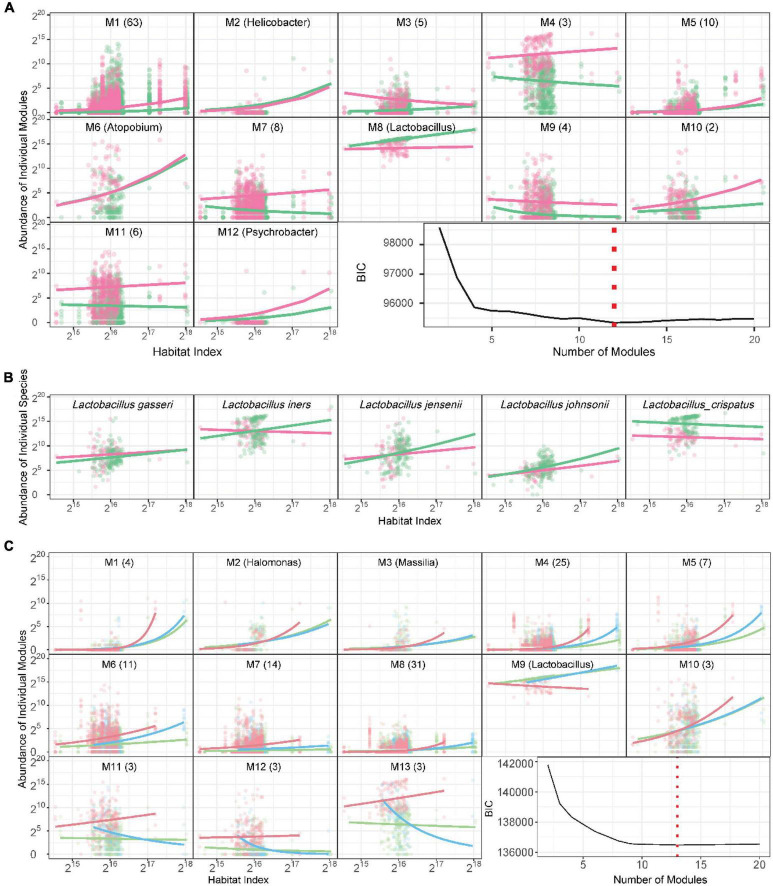
Functional clustering of genera into distinct modules. **(A)** BIC analysis identifies 12 functional modules, M1-M12, from 105 genera based on a bivariate functional clustering model incorporating the HI-varying pattern of microbial abundance in healthy (green) and AV groups (red). Shown in the paratheses are numbers of genera contained in a module. **(B)** Plots of abundance of the five most abundant *Lactobacillus* species against habitat index. Green and red lines represent the mean curves of HI-varying abundance changes for healthy and AV groups, respectively. **(C)** BIC analysis identifies 13 functional modules, M1-M13, from 105 genera based on a trivariate functional clustering model incorporating the HI-varying pattern of microbial abundance in the healthy category (pH = 3.8, green), the sub-healthy category (pH = 4.0–4.4, blue), and the AV category (pH = 4.6–5.4, red). Shown in the paratheses are numbers of genera contained in a module. Blue, green, and red lines represent the mean curves of HI-varying abundance changes for the three categories, respectively.

**Functional microbes:** We classify 104 identified genera and a mix of other unidentified genera into 12 modules M1–M12, each composed of functionally similar genera (Figure 3A and Supplementary Table 1). We find that M8 only contains *Lactobacillus*, confirming that this genus functions differently from other genera. In general, only three modules (25%), i.e., M2, M5, and M6, cannot be used to distinguish healthy and AV groups, whereas as many as 75% of modules can serve as predictors of AV risk, the majority of which are more abundant in the AV group than in the healthy group. From the plots of the abundance of the five richest species from *Lactobacillus* over HI, we find that only *L. crispatus* is favorable for a healthy vaginal ecosystem over a full range of HI, whereas *L. iners*, *L. jensenii* and *L. johnsonii* are favorable only at a high level of HI (Figure 3B).

An imbalance in vaginal microbial ecosystems can cause the alternation of pH values, thus, varying pH values may be associated with AV (Lin et al., 2021). We classify the pH-AV association into three categories, pH = 3.8 (healthy, *n* = 133), 4.0–4.4 (sub-healthy, *n* = 44), and 4.6–5.4 (AV risk, *n* = 66). We implement three-variate functional clustering (Wang et al., 2012) to classify 105 genera into distinct functional modules based on the similarity of how genera change their abundance with HI jointly under three categories of pH levels. We identify 13 modules, each containing a different number of genera and with a different HI-varying pattern (Figure 3C and Supplementary Table 2). All modules, except for M9, regardless of their number of genera, are more abundant in the AV category than in healthy and sub-healthy categories. Module M9 is only composed of one genus *Lactobacillus*, further confirming that *Lactobacillus* plays an important role in improving vaginal microbial ecosystems.

### Inferring context-specific idopNetworks

Individual microbes can serve as a predictor of AV risk at different levels of taxa and in terms of their functional discrepancies. The mechanistic role of individual microbes as a predictor can be better understood through microbial interaction networks. We reconstruct idopNetworks at the taxonomical (phylum) and functional levels.

**FIGURE 4:**
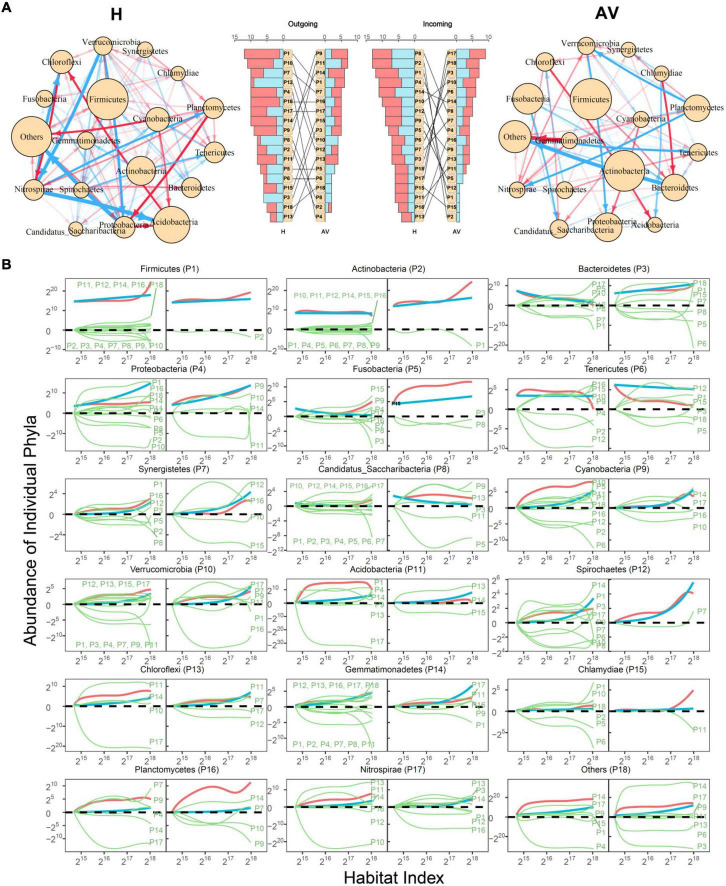
Taxonomic idopNetworks at the phylum level. **(A)** Network architecture showing the difference between healthy (H) and AV groups. Red and blue lines denote promotion and inhibition, respectively, with line thickness proportional to the strength of interactions. In the middle are the distributions of the number of outgoing links (red bars for up-regulation and blue for down-regulation) and incoming links (red bars for up-regulation and blue for down-regulation) across different phyla. **(B)** Decomposition of the net abundance trajectory (blue line) of each phylum, denoted as P1–P18, into its independent abundance trajectory (red line) and dependent abundance trajectory (green line) in the H group (left panel) and AV group (right panel). The names of phyla that regulate a given phylum are shown in the plot (positive regulators above the zero line and negative regulators above the zero line).

**Taxonomic networks:** Figure 4 illustrates 18-node bacterial idopNetworks among phyla for healthy and AV groups. We find that the taxonomic networks display remarkable discrepancies in topological architecture between the two groups (Figure 4A). The healthy network appears to be denser than the AV network due to a higher number of relatively weak links, suggesting that a healthy vagina can maintain a better balance between system function and stability. The number of outgoing links exerted by each phylum and the number of incoming links received by each phylum differ dramatically between the two networks (Figure 4A). These differences are also expressed in the relative number of positive and negative outgoing regulation and the relative number of positive and negative incoming regulation interactions for each phylum. For example, although existing as a predominant phylum in both networks, Firmicutes more actively regulates other phyla, with the number of outgoing links being up to one time larger in the healthy group than in the AV group. In particular, Firmicutes inhibits Bacteroidetes in the healthy vagina but promotes Bacteroidetes in the AV vagina. Actinobacteria is inhibited by only one phylum in the AV group but by many phyla in the healthy group.

Comparative analysis based on power fitting of Figure 2 shows that the transition of healthy to AV states is associated with increasing quantities of phyla, such as Actinobacteria, Bacteroidetes, Fusobacteria, and Tenericutes. It is interesting to note that these phyla each have a much higher level of independent abundance in the AV than in the healthy group (Figure 4B). Cyanobacteria and Acidobacteria are expressed, to a similar extent, in the healthy and AV groups, suggesting that they are neutral to health state. Yet, the independent abundance level of these two phyla reduces considerably from healthy to AV states. The level of independent abundance of a microbe is directly related to its intrinsic capacity, determining its fitness in a condition where it cannot derive any resources from its peers.

Phyla Actinobacteria, Bacteroidetes, Fusobacteria, and Tenericutes are inhibited by far fewer phyla in the AV group than in the healthy group (Figure 4B), which increases the likelihood that these microbes cause AV risk. On the other hand, phyla Cyanobacteria and Acidobacteria are promoted by many more phyla in the healthy group than in the AV group, increasing their capacity to maintain favorable ecological homeostasis in a healthy vagina. Taken together, idopNetworks provide a mechanistic interpretation of how different microbes at the phylum level interact with each other to bring about changes in the vaginal ecosystem from an eubiosis state to dysbiosis and vice versa.

**FIGURE 5:**
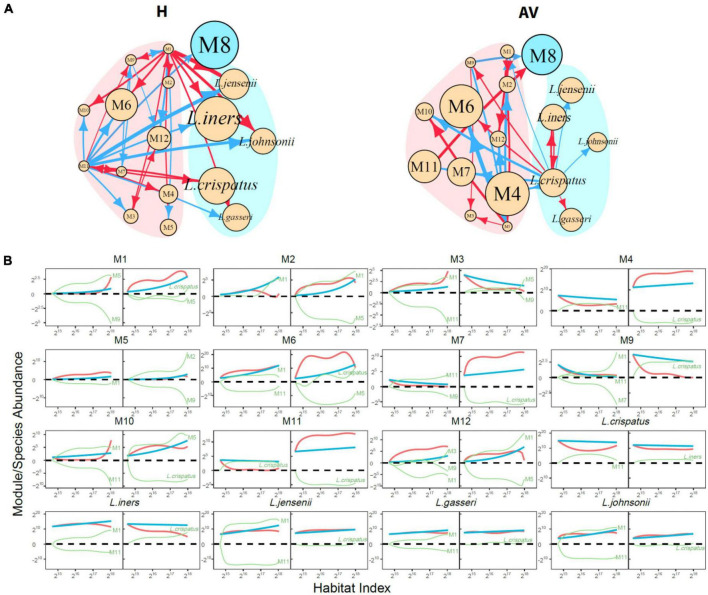
Functional idopNetworks among modules. **(A)** Network architecture showing the difference between healthy (H) and AV groups. Genus *Lactobacillus* is the only module contained in module M8, whose five most abundant species are added into the network. Red and blue lines denote promotion and inhibition, respectively, with line thickness proportional to the strength of interactions. **(B)** Decomposition of the net abundance trajectory (blue line) of each module into its independent abundance trajectory (red line) and dependent abundance trajectory (green line) in the H group (left panel) and AV group (right panel). The names of modules that regulate a given module are shown in the plot (positive regulators above the zero line and negative regulators above the zero line).

**Functional networks:** Figure 5 shows 12-node idopNetworks with functional modules for the healthy and AV groups. Considerable differences are found in network topology between two groups (Figure 5A). Module M8, composed of only genus *Lactobacillus* [predominant lactic acid bacteria (Gustafsson et al., 2011; Valenti et al., 2018)], has a considerably higher intrinsic capacity (described by independent abundance) than all other modules in the H network, but its intrinsic capacity reduces dramatically in the AV network. Unlike module M8, the intrinsic capacity of many other modules, especially M4, M6, M7, and M11, displays a pronounced increase from a healthy state to an AV state. The H network has two leaders, M1 and M11, which exert many outgoing links to other modules, but such leaders do not exist in the AV network (Figure 5A).

Figure 5B shows the decomposition of the net HI-varying change curve into its independent and dependent component curves for each module. M2, M5, and M6 have similar observed microbial abundance between different health states, but their underlying ecological mechanisms are different. M2 is promoted by M1 in the healthy group, but in the AV network although the former is still promoted, even to a larger extent, by the latter, the large independent component of M2 is counteracted by strikingly strong inhibition from M5. Similar interpretations can be made for M5 and M6.

The decomposition curves of Figure 5B can also mechanistically explain the reason why eight modules each have increased abundance in the AV group when compared to the healthy group. For example, M1 is activated by AV disease, displaying a large independent component, thus although it is inhibited by M5 and *L. crispatus*, its net abundance is still quite remarkable in the AV group. The independent component of M4 is strikingly larger in the AV group than in the healthy group, but although promoted by M11 in the AV group and inhibited by *L. crispatus* in the AV group, the net abundance of M4 is still much larger in the latter than in the former. Although M12’s independent component is virtually very large in the healthy group, its net abundance is reduced because it is strongly inhibited by other modules. Yet, despite its smaller independent component in the AV group, M12 is promoted by M1 and inhibited by M5, ultimately making M12’s net abundance level larger in the AV group than in the healthy group.

As an important module, M8, i.e., *Lactobacillus*, we identify its five most abundant species to characterize the detailed role of each species in mediating network change from a healthy state to an AV state. As seen from the power fitting (Figure 3B), *L. crispatus* is consistently much richer in the healthy group than in the AV group. The independent abundance level of all species, especially *L. crispatus* and *L. iners*, reduces from a healthy state to an AV state, showing that these species participate in shifting vaginal ecosystems from symbiosis to dysbiosis (France et al., 2016). In the H network, all five species are promoted or inhibited by other modules, and none of them exerts outgoing links, neither to each other nor to any other microbes from other modules (Figure 5A). Just as *Lactobacillus* is one of the most important genera (Wang et al., 2012; Valenti et al., 2018), *L. crispatus* is one of the most important species in this genus, which is favorable to maintain a heathy vaginal state (France et al., 2016). It is interesting to note that *L. crispatus* is only one abundant species that establishes a mutualistic cycle with module M11. Although M11, containing nine infrequent genera, *Enterococcus*, *Gemella*, *Mageeibacillus*, *Megasphaera*, *Mycoplasma*, *Peptoniphilus*, *Phyllobacterium*, *Staphylococcus*, and *Veillonella*, are unfavorable to vaginal health, it simultaneously serves as an inhibitor of other unfavorable microbes (such as M3, M6, M10, etc.) and as a promotor of the favorable *L. crispatus* in the H network (Figure 5B). In the AV network, none of *Lactobacillus*’ five species receives any incoming link from other modules, suggesting that their growth is purely dependent on their own capacity. *L. crispatus* is a primary leader, exerting numerous outgoing links, not only in the subnetwork composed of its species counterparts, but also in the entire functional network (Figure 5A). Because of these multifaceted roles in the AV network, *L. crispatus*’ capacity to exploit and digest resources for symbiotic maintenance is largely weakened. This may be one important cause or consequence of AV.

In addition, *L. crispatus*, as a favorable species, unexpectedly promotes some unfavorable modules, such as M6 and M9 (Figure 5B) and also promotes its peers *L. iners* and *L. gasseri*. As seen from the power fitting (Figure 3B), these peers are not always favorable for healthy vagina ecosystems; in some cases, they are positively associated with AV. The independent abundance of *L. iners* is reduced in the AV group, but its observed abundance is augmented from promotion from *L. crispatus* (Figure 5B). *L. jensenii*, *L. gasseri*, and *L. johnsonii* each are promoted by M1, but inhibited to a larger extent by M11, in the H network, whereas each of these three species is only promoted by *L. crispatus* in the AV network (Figure 5B).

### Tracing topological changes of idopNetworks across pH gradients

**FIGURE 6:**
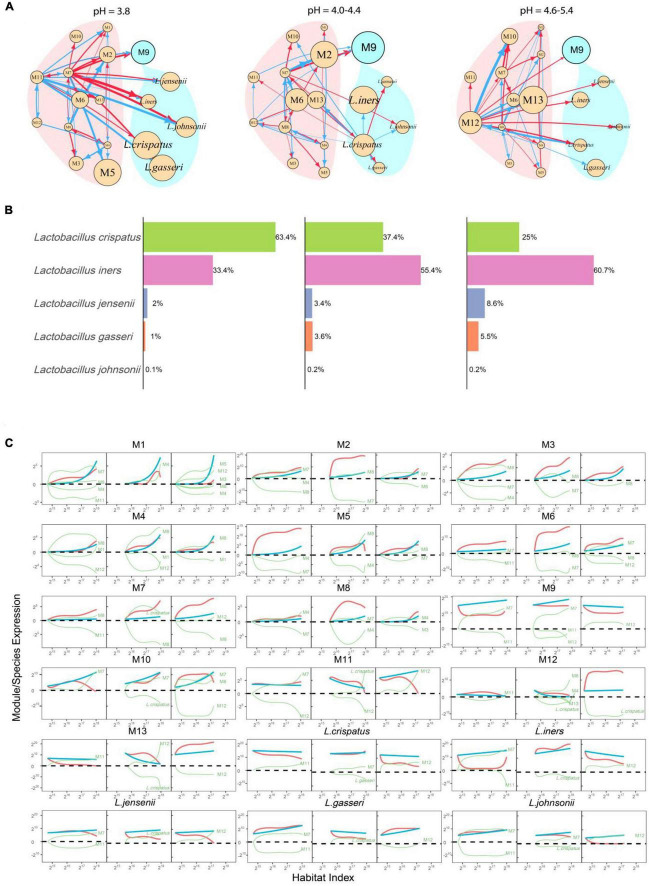
PH-dependent functional idopNetworks among modules. **(A)** Network architecture showing differences among the healthy category (pH = 3.8), the sub-healthy category (pH = 4.0–4.4), and the AV category (pH = 4.6–5.4). Genus *Lactobacillus* is the only module contained in module M9, whose five most abundant species are added into the network. Red and blue lines denote promotion and inhibition, respectively, with line thickness proportional to the strength of interactions. **(B)** The percentages of abundance of the five most abundance species of *Lactobacillus* in this genus in three pH categories. **(C)** Decomposition of the net abundance trajectory (blue line) of each module into its independent abundance trajectory (red line) and dependent abundance trajectory (green line) in the healthy category (left panel), the sub-healthy category (middle panel), and the AV category (right panel). The names of modules that regulate a given module are shown in the plot (positive regulators above the zero line and negative regulators above the zero line).

We reconstruct 13-node functional idopNetwork for different pH categories (Figure 6). Each module displays pH-dependent differences in HI-varying abundance curves (Figure 3C), and these differences can be mechanistically explained by the networks (Figure 6A). For example, M11-M13 are observed to be much more abundant in the AV category than in healthy and sub-healthy categories. From the decomposition curves of Figure 6C, we can see that some of these differences (such as M7, M10, M12 and M13) are due to increasing independent abundance when the vaginal environment becomes alkaline, whereas some, such as M2, M3, M5, M6, M8, and M11) result strong inhibition from other modules in healthy and sub-healthy vaginas.

It is interesting to see that as a whole, *Lactobacillus* has a greater independent component in the AV category than in the healthy category (Figure 6C). However, *Lactobacillus* is more strongly promoted by M7 in the healthy vagina than M12 in the diseased vagina, making its overall abundance level higher in the former than in the latter. During the transition from healthy to sub-healthy state, the intrinsic reproductive capacity of *Lactobacillus* is strengthened, but because of new inhibition from M12, its overall abundance is reduced.

Considering their unique role in transiting the vagina from a healthy state to AV by maintaining its subacidity, we choose the five most abundant species of *Lactobacillus*, including *L. crispatus*, *L. iners, L. gasseri*, *L. jenseni*, and *L. johnsonii*, to be added into pH-varying functional networks (Figure 6A). We analyzed the proportions of these five species (Figure 6B). *L. crispatus* and *L. iners* are two predominant species, together occupying 90% of genus *Lactobacillus* in the healthy vagina, but they are different in the isomers of lactic acid they produce as end products of fermentation. *L. crispatus* can produce L- and D-lactic acid, whereas *L. iners* can only produce L-lactic acid (Amabebe and Anumba, 2018). We find that from categories 1 to 3, *L. crispatus* decreases its population proportion in order: 63.3%—37.4%—25.0%, whereas *L. iners* increases its population proportion in order: 33.4%—55.4%—60.7%, suggesting that the relative abundance of these two species is a predictor of AV risk. An increasing proportion of *L. crispatus* to *L. iners* is favorably associated with the healthy state. It is possible that 61% is a threshold for reciprocal transition between health and AV; i.e., if *L. crispatus* reaches 61% or higher of the microbiotic population, the vaginal microbiota maintains a healthy environment, whereas if *L. iners* reaches 61% or higher, the vaginal microbiota are dysbiotic.

Why does the relative proportion of *L. crispatus* vs. *L. iners* decrease from the healthy to the AV state? This can be explained from the structure of idopNetworks. *L. crispatus* exhibits a larger independent component in a more acid vagina than in a more alkaline vagina, whereas an inverse pattern is found for *L. iners* (Figure 6C). Although *L. crispatus* in both conditions is promoted by a module, M11 in the acid condition and M12 in the alkaline condition, this regulation is unidirectionally commensalistic in the former but bidirectionally altruistic/predatory in the latter. Thus, while M12 leads to the increasing abundance of *L. crispatus*, this increase quickly inhibits the existence of M12, reducing its capacity to promote *L. crispatus*. Although *L. iners* receives promotion from a module in both conditions, it is simultaneously inhibited by the other module in a healthy vagina. For this reason, the increase of *L. iners*’ abundance is limited when vaginal pH level is more acid.

### Vagina idopNetwork correlates with changes of menstrual cycles

**FIGURE 7:**
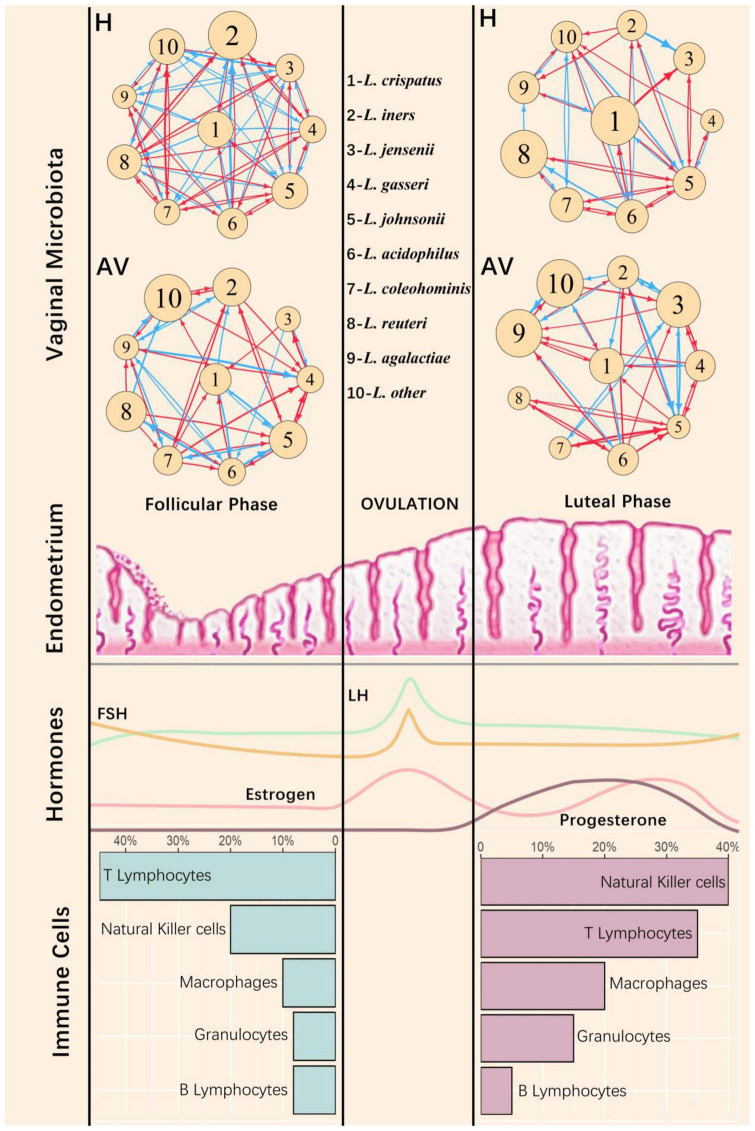
Vaginal microbial networks in response to physiological changes of the endometrium during menstruation cycle. The repetitive cycles of the human endometrium among the follicle, ovulation, and luteal phases are accompanied by morphological changes and cyclical fluctuations of sex hormones [including estrogen, progesterone, follicle hormone (FSH), and luteinizing hormone (LH)] as well as different types of immune cells (Agostinis et al., 2019). At the top of the figure are the microbial networks composed of the nine most abundant Lactobacillus species (denoted as 1–9) and the remaining microbes (other) for healthy (H) and AV groups. The nodes of the networks represent the independent abundance components of individual microbes, with the size of circles proportional to the value of such components. The edges of the networks are directional promotion (red) or inhibition (blue) between a pair of microbes.

A women’s menstrual cycle includes three cyclic stages, the follicular phase, the luteal phase, and the menstrual phase with different endometrial characteristics in each phase. The follicular and luteal phases are facilitated by follicle-stimulating hormone (FSH) and luteinizing hormone (LH), respectively. In the follicular phase, estrogen-dominant hormone mediates the regeneration of the functional layer of the endometrium, whereas progesterone drives the endometrium to undergo various changes in preparation for embryo implantation in the luteal phase. These phase-dependent hormonal changes in the endometrium are associated with alterations in the proportion of different types of immune cells (Figure 7). We find that microbiota flora in the vagina vary with the shift of physiological states in the upper reproductive tract (Figure 7).

We reconstruct idopNetworks among the nine most abundant species of *Lactobacillus* with all remaining microbes treated as others in the vagina, separately for the follicular and the luteal phases (Figure 7). In the healthy group, the interconnection density of the network composed of *Lactobacillus* species reduces dramatically from follicular to luteal phases, whereas such a phase-dependent change does not occur for the AV group. At the follicular phase, the intrinsic capacity of *L. crispatus* to expand its abundance reduces from the healthy to the unhealthy state, but this capacity stays stable over health state in the luteal phase. *L. iners* reduces its intrinsic capacity for reproduction from the healthy to unhealthy states to a greater extent at the luteal than the follicular phases. For the healthy group, *L. crispatus* reduces its intrinsic growth capacity, accompanied by the increase of *L. iners*’ intrinsic capacity, from follicular to luteal phases, whereas the intrinsic growth capacity of both species does not markedly change between two phases. Taken together, the *Lactobacillus* network is more adaptive in its overall topological features, especially the relative intrinsic growth capacity of two key species, *L. crispatus* and *L. iners*, as a function of physiological change to the endometrium. Also, while *L. crispatus* is a key determinant of AV risk at the follicular phase, this determinant is replaced by *L. iners* at the luteal phase.

## Discussion

Most studies linking microbiota with natural and health processes focus on comparing the relative abundance of individual microbes between different regimes. By comparing the 10 major phyla identified in the vaginal ecosystem, Wang et al. (2020) found that Firmicutes (mainly *Lactobacillus*) predominates the vaginal microbiota in healthy women, whereas Actinobacteria and Bacteroidetes became much more abundant in women infected by AV. The role of *Lactobacillus* is speculated to include a reduction in the microenvironmental pH level, generating various bacteriostatic and bactericidal compounds, and competitively excluding other bacterial species (Wang et al., 2012; Valenti et al., 2018). However, without a mechanistic picture of how individual microbes, such as Firmicutes, Actinobacteria, and Bacteroidetes, mediate the occurrence of AV, the precise treatment of this disease by probiotic supplementary agents remains problematic. For example, *L. crispatus* is a key species of *Lactobacillus* to meliorate vaginal dysbiosis, but because it also plays a role in inhibiting the other microbes that promote it, probiotics containing this species requires a balance of multiple microbes to maximize its efficacy.

In this article, we used a powerful network tool to dissect how each microbe interacts with all other microbes to determine AV, providing a unique way to predict AV risk by understanding the mechanisms underlying microbiota-host crosstalk. Our predictive model includes three hierarchical stages, the calculation of HI, allometric scaling fitting of individual microbe levels, and microbial interaction modeling by qdODEs. As compared to the healthy group, the HI of the AV group reduces only slightly (Figure 1), suggesting that the reduction of the total amount of microbes does not fully reflect AV risk. Yet, by plotting the abundance level of individual microbes at a specific level of taxon, a set of microbes that distinguish between healthy and AV groups can be identified. For example, Firmicutes are more abundant in healthy women than in non-healthy women, whereas Actinobacteria, Bacteroidetes, and Fusobacteria display an increasing abundance level in AV women (Figure 2A). The association between microbial abundance and AV can be more clearly dissected at the genus level; a striking decrease in *Lactobacillus* and a striking increase in multiple aerobes, such as genera *Streptococcus*, *Aerococcus*, etc., accompanies the onset of AV (Figure 2B). Taken together, the slope of allometric scaling curves for certain genera that change their abundance with HI can be used as a powerful predictor of AV risk.

The network model provides a mechanistic understanding of predictive models for AV risk. In a highly dense bioenvironment, such as the vagina, the function of any single microbe is regulated by other microbes (Ravel et al., 2011; Chen et al., 2021). The transition of vaginal microflora from a healthy (symbiotic) to abnormal (dysbiotic) state is not only characterized by the change of abundance of individual key microbes, but also through the interaction networks of all microbes as a cohesive whole. We reconstruct taxonomic microbial networks using natural taxa of microbes as network nodes, and find that microbes are not fully interconnected at the phylum level in symbiotic vaginal microflora, but with a density being higher than that in dysbiotic vaginal microflora (Figure 3). There has been a long debate on the complexity-stability relationship in living systems (Valenti et al., 2018). One view suggests that complex communities enhance community stability (Gravel et al., 2016; Goyal et al., 2022; Yonatan et al., 2022). However, this view is challenged by May (1973) who used mathematical models to find the positive association of community destabilization and complexity. The two distinct views implies that community stability is not related to community complexity in a linear way, rather their relationship is non-linear. The non-linear hypothesis well explains our discovery; i.e., microbial links in the vaginal ecosystem are maintained at a threshold level, below or above which vaginal microflora becomes abnormal.

More importantly, we find that the decrease of abundance in Firmicutes is due to both intrinsic and extrinsic factors, i.e., the reduction of its carrying capacity (described by the independent component) and the negative regulation it receives from Actinobateria (described by the dependent component) (Figure 3B). In the healthy vagina, Firmicutes is simultaneously upregulated and downregulated by multiple microbes and, ultimately, receives slight overall positive regulation after regulation in different signs is canceled out. This result suggests that, in order to improve the dysbiosis of vaginal microflora through Firmicutes, using probiotics that only contain this microbe is not sufficient, rather it should be mixed with positive regulators.

Several phyla, such as Actinobacteria, Bacteroidetes, Proteobacteria, etc., are activated, exhibiting a striking increase in their independent abundance, in abnormal vaginal microflora. As such, specific medications can be designed to control these microbes so that the vaginal microenvironment can be improved. This strategy may be efficient for Actinobacteria because it is only downregulated by Firmicutes. Yet, for the other phyla that are regulated by many different regulators, special attention should be paid to the development of medications that can balance the co-occurrence of the regulatees and regulators. For example, to control Proteobacteria, its positive regulators, Cyanobacteria and Verrucomicrobia, should be controlled simultaneously as a whole. Our idopNetworks chart a detailed roadmap of how and how much each phylum regulates, or is regulated by, every other phylum across subjects (Figure 3B), from which an optimal strategy to control specific microbes can be designed and delivered.

We have also reconstructed functional microbial networks with functional units as network nodes. Although microbes from different taxa are phylogenetically different, they may perform a similar function. This allows us to classify different microbes at different taxonomic levels into distinct functional modules. This classification has two advantages. First, functional networks based on these modules can better explain the mechanistic relationships among microbes and their impact on disease outcome. Second, it provides a way to reconstruct networks from high-dimensional data. Clinically more useful and informative microbial networks should be reconstructed with a fine-grained unit, such as genera, species, strains, or even genes. However, such networks will have too many nodes to be coded into a graph because of computational burden and instability. The classification produces different modules, each with a smaller number of entities that make it possible to reconstruct networks. We classify 104 genera identified in healthy and AV-infected women into multiple modules based on the similarity of HI-varying abundance change over different health states and vaginal pH gradients. Results from functional networks based on modules well support those from taxonomic networks at the phylum level, while providing an additional insight into microbial interactions and their impact on AV risk. In both functional clustering over health states (heathy vs. AV) and pH levels (low, middle, high), genus *Lactobacillus* is always attributed to a module that only is composed of this genus itself. This result reflects the unique role of *Lactobacillus*, one of the most often found inhabitants in the vagina, in maintaining the vaginal ecosystem of healthy women (Goyal et al., 2022). Analysis of functional networks shows that *Lactobacillus* is positively regulated by the other functional module containing many species in healthy vaginal microbiota, but this positive regulation is largely weakened when the vaginal ecosystem become dysbiotic (Figure 6). This is accompanied by an increasingly alkaline microenvironment.

By further dissecting the role of *Lactobacillus* at its species level, this genus is predominated by two species, *L. crispatus* and *L. iners*, with its next three most abundant species together accounting for only 3–14% of the total population (Figure 6B). Compared to *L. iners*, *L. crispatus* has a greater capacity to produce lactic acids that break down carbohydrates for energy when oxygen levels are low. In healthy vaginas, *L. crispatus* is promoted by other microbes, but it turns to serve as a regulator to regulate other microbes, even including promoting unfavorable microbes, in less healthy vaginas. These multiple tasks would, with no doubt, affect its role in maintaining vaginal symbiosis. Also, when a vagina becomes alkaline, *L. crispatus* receives positive regulation from, but while exerting inhibition to, the same microbial module. This aggressive/altruistic relationship forms a paradox for *L. crispatus*’ role in maintaining vaginal symbiosis and alleviating vaginal dysbiosis.

In the healthy vagina at a normal pH, *L. crispatus* and *L. iners* account for about 63% and 33% of the total genus mass, respectively. This relative proportion is in agreement with the golden dissection hypothesis of animal conflict (Wang et al., 2019; He et al., 2021; Wu et al., 2021), with which the more abundant *L. crispatus* (>61%) tends to cooperate with the less abundant *L. iners* (<38%). This relative proportion changes when pH values increase. In the abnormal vagina at a more alkaline pH, the proportions of *L. crispatus* and *L. iners* become 25 and 60.7%, respectively. This proportion suggests the surrender-resistance hypothesis of animal conflict (Wu et al., 2021), by which the less abundant *L. crispatus* would be sacrificed if it chooses to cooperate, but it may benefit from its choice of conflict with the more abundant *L. iners*. In either case, the two species tend to compete against each other, leading the vaginal ecosystem to be dysbiotic (Pacha-Herrera et al., 2022).

We report a detailed application of idopNetworks as a mechanistic predictor of AV risk. It is not surprising that this application produces interpretable results about the interactive mechanisms underlying the microecological balance of vaginal microflora, given that the development of this model was derived from the integration of biologically meaningful evolutionary game theory and prey-predator equation (Chen et al., 2019, 2022; Griffin et al., 2020; Wu and Jiang, 2021). The results from this application could be potentially more useful for the design of effective medications to treat AV when a more sample size is used and when more powerful statistical solution for curve fitting and stochastic modeling is developed. At the same time, the modified model can be used to reveal other microbial community assembly, such as the gut microbiota, and their impacts on health and natural processes.

## Data availability statement

The original contributions presented in the study are included in the article/Supplementary material, further inquiries can be directed to the corresponding authors.

## Ethics statement

The studies involving human participants were reviewed and approved by Tianjin Medical University General Hospital. The patients/participants provided their written informed consent to participate in this study.

## Author contributions

QW, FX, and RW conceived of the project. AD and JZ performed data analysis. CW provided the data. ChG and ClG critically reviewed the manuscript. QW and RW wrote the manuscript. All authors contributed to the article and approved the submitted version.

## Supplementary material

Supplementary Table 1 Twelve modules across healthy and AV groups.

Supplementary Table 2 Thirteen modules across three pH levels.
