# Supplementary material for: Vaginal microbiota networks as a mechanistic predictor of aerobic vaginitis
Source: Front Microbiol. 2022 Oct 20;13:998813. doi: 10.3389/fmicb.2022.998813 (PMC9631484; doi:10.3389/fmicb.2022.998813)
Supplement: Supplementary Table 2 — Thirteen modules across three pH levels. [file Data_Sheet_1.PDF]

	M1	M2	M3	M4	M5	M6	M7	M8
1	Helicobacte	Halomonas	Massilia	Actinomyce	Acinetobac	Anaerococc	Arcanobact	Actinomyce
2	Neisseria	NA	NA	Alistipes	Bacillus	Corynebact	Fenollaria	Anoxybacil
3	Psychrobac	NA	NA	Arthrobact	Bacteroides	Dialister	Geobacter	Bradyrhizol
4	Shewanella	NA	NA	Blautia	Clostridium	Enterococc	Mageeibac	Bulleidia
5	NA	NA	NA	Carnobacte	Faecalibact	Escherichia	Megasphae	Campyloba
6	NA	NA	NA	Fusobacter	Pediococcu	Eubacteriu	Mesorhizol	Chlamydia
7	NA	NA	NA	Granulicate	Roseburia	Finegoldia	Mobiluncus	Filifactor
8	NA	NA	NA	Haemophil	NA	Gemella	Mycoplasm	Halospiruli
9	NA	NA	NA	Janibacter	NA	Klebsiella	Peptoniphi	Hyphomicr
10	NA	NA	NA	Lachnoclos	NA	Staphyloco	Peptostrep	Lachnospira
11	NA	NA	NA	Lactococcu	NA	Veillonella	Phyllobacte	Limnohabit
12	NA	NA	NA	Lysobacter	NA	NA	Porphyrom	Luteimonas
13	NA	NA	NA	Megamona	NA	NA	Pseudonoc	Methyloter
14	NA	NA	NA	Oceanobac	NA	NA	Pseudoram	Nitrospira
15	NA	NA	NA	Oscillospira	NA	NA	NA	Nocardioid
16	NA	NA	NA	Paenibacill	NA	NA	NA	Oribacteriu
17	NA	NA	NA	Parabacter	NA	NA	NA	Polaromon
18	NA	NA	NA	Peptococcu	NA	NA	NA	Pseudoxan
19	NA	NA	NA	Planococcu	NA	NA	NA	Rhizobium
20	NA	NA	NA	Ramlibacte	NA	NA	NA	Rhodanoba
21	NA	NA	NA	Ruminococ	NA	NA	NA	Rhodoplan
22	NA	NA	NA	Sphingomo	NA	NA	NA	Rubrobacte
23	NA	NA	NA	Stenotroph	NA	NA	NA	Sphingomo
24	NA	NA	NA	Vibrio	NA	NA	NA	Steroidoba
25	NA	NA	NA	Yersinia	NA	NA	NA	Streptomyc
26	NA	NA	NA	NA	NA	NA	NA	Sutterella
27	NA	NA	NA	NA	NA	NA	NA	Tannerella
28	NA	NA	NA	NA	NA	NA	NA	Thiobacillus
29	NA	NA	NA	NA	NA	NA	NA	Tissierella
30	NA	NA	NA	NA	NA	NA	NA	Varibaculu
31	NA	NA	NA	NA	NA	NA	NA	Virgibacillu

M9	M10	M11	M12	M13
Lactobacill	Atopobium	Aerococcus	Alloscardov	Gardnerella
NA	Others	Prevotella	Bifidobacte	Streptococcus
NA	Pseudomo	Ureaplasm	Sneathia	NA
NA	NA	NA	NA	NA
NA	NA	NA	NA	NA
NA	NA	NA	NA	NA
NA	NA	NA	NA	NA
NA	NA	NA	NA	NA
NA	NA	NA	NA	NA
NA	NA	NA	NA	NA
NA	NA	NA	NA	NA
NA	NA	NA	NA	NA
NA	NA	NA	NA	NA
NA	NA	NA	NA	NA
NA	NA	NA	NA	NA
NA	NA	NA	NA	NA
NA	NA	NA	NA	NA
NA	NA	NA	NA	NA
NA	NA	NA	NA	NA
NA	NA	NA	NA	NA
NA	NA	NA	NA	NA
NA	NA	NA	NA	NA
NA	NA	NA	NA	NA
NA	NA	NA	NA	NA
NA	NA	NA	NA	NA
NA	NA	NA	NA	NA
NA	NA	NA	NA	NA
NA	NA	NA	NA	NA
NA	NA	NA	NA	NA
NA	NA	NA	NA	NA
NA	NA	NA	NA	NA
